# Cep215 is essential for morphological differentiation of astrocytes

**DOI:** 10.1038/s41598-020-72728-7

**Published:** 2020-10-12

**Authors:** Donghee Kang, Wonjung Shin, Hyunjeong Yoo, Seongjae Kim, Seongju Lee, Kunsoo Rhee

**Affiliations:** 1 Department of Biological Sciences, Seoul National University, Seoul, 08826 Korea; 2 Department of Anatomy, Inha University School of Medicine, Incheon, 22212 Korea

**Keywords:** Cell biology, Neuroscience

## Abstract

Cep215 (also known as Cdk5rap2) is a centrosome protein which is involved in microtubule organization. Cep215 is also placed at specific subcellular locations and organizes microtubules outside the centrosome. Here, we report that Cep215 is involved in morphological differentiation of astrocytes. Cep215 was specifically localized at the glial processes as well as centrosomes in developing astrocytes. Morphological differentiation of astrocytes was suppressed in the *Cep215*-deleted P19 cells and in the Cep215-depleted embryonic hippocampal culture. We confirm that the microtubule organizing function of Cep215 is critical for the glial process formation. However, Cep215 is not involved in the regulation of cell proliferation nor cell specification. Based on the results, we propose that Cep215 organizes microtubules for glial process formation during astrocyte differentiation.

## Introduction

Microtubule is an important building block for cellular morphogenesis. During neurogenesis, microtubules are nucleated from the centrosome in early phase^1^. However, in late phase, the centrosome loses its microtubule organizing activity as soon as it loses pericentriolar material (PCM) proteins^1^. Instead, non-centrosomal microtubules play critical roles for neuronal morphogenesis^2^. It is known that microtubule nucleating factors, such as Tpx2 and the Haus complex, locally regulate microtubule nucleation outside the centrosome^3,4^. Non-centrosomal microtubule organization is also observed in other differentiated cells as well^5,6^.

Cep215 (also known as Cdk5rap2) is a major PCM protein with at least two functional domains: CM1 is essential for interaction with γ-tubulin ring complex (γ-TuRC)^7^ and CM2 is essential for interaction with specific centrosome proteins, such as pericentrin and Akap450^8^. It is also known that Cep215 can interact with other cellular proteins^7^. The structural information suggests that Cep215 is a recruiter of γ-TuRC to specific loci, including the centrosome^6^. For example, Cep215 forms complex with the microtubule plus-end tip and organizes microtubules at the cell periphery^9,10^. *Centrosomin* (*Cnn*), a *Drosophila* ortholog of *Cep215*, can be alternatively spliced to generate testis-specific isotype protein, Cnn-T^11^. Cnn-T includes a mitochondria-binding domain instead of the centrosome binding domain and organizes microtubules at the mitochondria in *Drosophila* sperm^11^.

Mutations in *CEP215* have been reported among microcephaly patients^12–14^. *Cep215* mutations in mice are similar to the human conditions with reduced brain size and a strikingly thin neocortex already at early stages of neurogenesis^15^. Depletion of Cep215 in mouse embryo brain revealed precocious neurogenesis with an increase in cell cycle exit^16^. The *Hertwig’s anemia* (*an*) mouse which has a genomic inversion at exon 4 of *Cep215* was previously reported a macrocytic, hypoproliferative anemia and leukopenia with a high level of spontaneous aneuploidy^17^. Microcephaly was also observed in the *Cep215*^*an/an*^ mice^18^. Mutations in *Cnn* promoted extra branching of dendrite via regulation of the microtubule nucleation at the Golgi complex of specific *Drosophila* neurons^19^. Therefore, Cep215 is critical for proliferation and differentiation of neuronal progenitor cells as well as of other stem cells.

Brain tissues consist of neurons and glial cells both of which are originated from radial glial cells. Astrocyte, a major glial cell, contains a small soma, extensive branches and fine processes with a unique intermediate protein, glial fibrillary acidic protein (Gfap)^20^. Gfap, a building block of astrocyte processes, is known to move along the microtubules^21^. However, it remains to be understood how microtubules contribute to morphogenesis of astrocytes.

In this study, we investigated the involvement of Cep215 in glial process formation during astrocyte differentiation. Our results revealed that Cep215 is located at the glial processes as well as centrosomes and plays an essential role in glial process formation by regulation of microtubule organization.

## Results

### Cep215 expression in differentiated P19 cells

**Figure 1:**
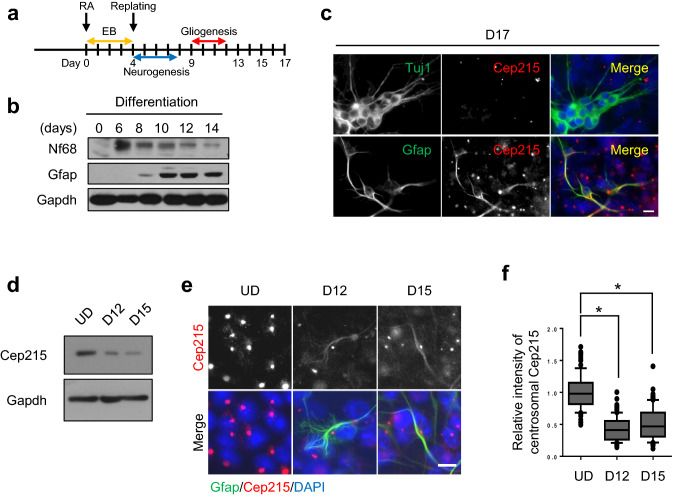
Subcellular localization of Cep215 in P19 cells under differentiation. (**a**) Experimental scheme of glial differentiation of P19 cells. The cells were treated with retinoic acid (RA) for 4 days to induce embryoid body (EB) and cultured for up to 17 days. Neurogenesis occurs at the early stage of differentiation (D4-8) whereas gliogenesis occurs at the late stage of differentiation (D9-13). (**b**) P19 cells were treated with RA for differentiation and subjected to immunoblot analysis with antibodies specific to Nf68, Gfap and Gapdh. (**c**) The P19 cells at D17 were subjected to coimmunostaining analysis with antibodies specific to Cep215 (red) along with Tuj1 or Gfap (green). (**d–f**) Undifferentiated (UD) and differentiated (D12 and D15) P19 cells were subjected to immunoblot (**d**) and coimmunostaining (**e**) analyses with antibodies specific to Cep215, Gapdh and Gfap. (**f**) Intensities of the centrosomal Cep215 signals were measured. In case of D12 and D15, only Gfap-expressing cells were subjected to analysis. Greater than 90 cells per experimental group were estimated in three independent experiments. The statistical significance was analyzed using one-way ANOVA. *P < 0.05. (**c**, **e**) Nuclei were stained with DAPI (blue). Scale bars, 10 μm.

We used P19 mouse embryonic carcinoma cells to examine importance of Cep215 during neurogenesis. Upon stimulation of retinoic acid (RA), P19 cells differentiate into neurons and glial cells at early and late stages, respectively^22^ (Fig. 1a). Immunoblot analyses revealed that neurofilament 68 (Nf68), a neuronal marker, was detected at as early as day 6, whereas Gfap, an astrocyte marker, started to be expressed at day 8 after the RA treatment (Fig. 1b). We performed immunostaining analyses to determine subcellular localization of Cep215 in differentiated P19 cells. Although there were some variations with the centrosomal Cep215 signals by cell types, specific Cep215 signals were detected at the centrosomes of all cells as expected (Fig. 1c). In the Tuj1-positive cells, the centrosomal and the cytoplasmic Cep215 signals were hardly observed. To our surprise, specific Cep215 signals were also detected at glial processes of astrocytes along with the strong signals at the centrosomes (Fig. 1c). The total protein levels as well as the centrosomal signals of Cep215 were higher in undifferentiated, dividing P19 cells (Fig. 1d–f). Although the expression of Cep215 was reduced after induction of differentiation, astrocyte-differentiated P19 cells quietly maintained centrosomal Cep215 level. In addition, cytoplasmic distribution of Cep215 started to appear at day 12 of glial differentiation (Fig. 1e). These results suggest that Cep215 might be involved in morphological differentiation of astrocytes.

### Suppression of astrocyte differentiation in *Cep215*-deleted P19 cells

**Figure 2:**
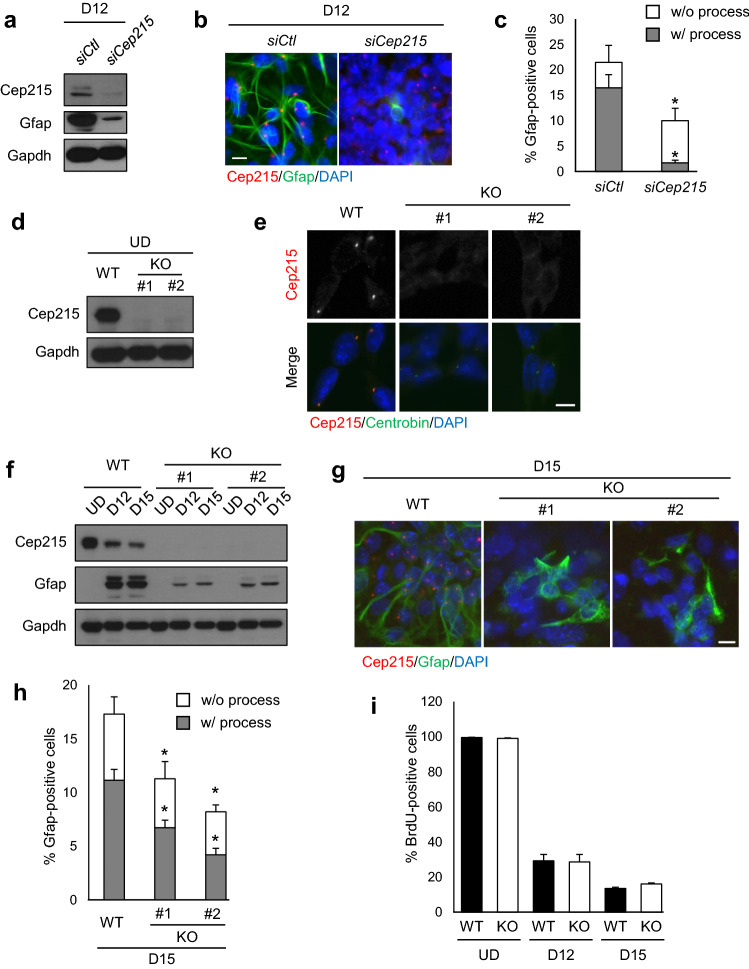
Defects in glial differentiation of the *Cep215*-deleted P19 cells. (**a-c**) Cep215 in P19 cells was depleted with siRNA transfection. The cells at D12 were subjected to immunoblot (**a**) and coimmunostaining (**b**) analyses with antibodies specific to Cep215, Gfap and Gapdh. (**c**) The number of Gfap-positive cells with or without glial processes were counted. (**d**, **e**) *Cep215* in P19 cells was deleted with the CRISPR-Cas9 method. Two *Cep215*-deleted cell lines (#1 and #2) were subjected to immunoblot (**d**) and coimmunostaining (**e**) analyses. (**f**, **g**) The *Cep215*-deleted (KO #1 and KO #2) P19 cells were differentiated (D12 and D15) and subjected to immunoblot (**f**) and coimmunostaining (**g**) analyses with antibodies specific to Cep215, Gfap and Gapdh. (**h**) The number of Gfap-positive cells with or without glial processes were counted at D15 of differentiation. (**i**) Undifferentiated (UD) and differentiated (D12 and D15) cells were subjected to BrdU incorporation assay. The number of BrdU-positive cells at the indicated days were counted. (**b**, **e**, **g**) Nuclei were stained with DAPI (blue). Scale bars, 10 μm. (**c**, **h**, **i**) Greater than 400 cells per experimental group were counted in three independent experiments. The statistical analysis was performed by unpaired *t*-test (**c**, **i**) and two-way ANOVA (**h**). Error bars, SEM. *P < 0.05.

To investigate importance of Cep215 during astrocyte differentiation, we transfected a specific siRNA (*siCep215*) into P19 cells. Depletion of Cep215 was confirmed with immunoblot and coimmunostaining analyses (Fig. 2a,b). At the same time, we observed a significant reduction of the cellular Gfap levels in Cep215-depleted cells (Fig. 2a). The immunostaining analysis revealed that the number of Gfap-expressing cells was significantly reduced after the Cep215 depletion (Fig. 2b,c). Furthermore, Cep215-depleted cells hardly generated glial processes (Fig. 2b,c). These results suggest that Cep215 is necessary for Gfap expression and for glial process formation during astrocyte differentiation.

We deleted *Cep215* in P19 cells, using the CRISPR-Cas9 method. Immunoblot and immunostaining analyses confirmed the absence of Cep215 signals in the knockout cell lines (Fig. 2d,e). When glial differentiation was induced in the *Cep215*-deleted P19 cells, we confirmed that Gfap expression was significantly reduced (Fig. 2f,g). Furthermore, the number of cells with glial processes was also significantly reduced (Fig. 2g,h). These phenotypes were observed in two different knockout cell lines (Fig. 2f–h). These results indicate that Cep215 is essential for astrocyte differentiation. When *Cep215*-deleted P19 cells were cultured in a neuronal differentiation condition, they differentiated into neurons as effectively as the control cells did, suggesting that Cep215 is not critical for neuronal differentiation at least at early stages. (Supplementary Fig. 1). Rather Cep215 may be involved in branching formation of neuronal dendrites^19,23^.

Gfap expression would be reduced if the *Cep215*-deleted cells proliferated less than the control cells. To rule out the possibility, we determined the proliferation activity of *Cep215*-deleted cells using bromodeoxyuridine (BrdU) incorporation assays. We treated *Cep215*-deleted P19 cells with BrdU for 24 h and counted the number of BrdU-positive cells. As expected, the number of BrdU-positive cells decreased after glial differentiation (Fig. 2i, Supplementary Fig. 2). However, we did not observe any difference in the proliferation activities of the wild type and *Cep215*-deleted cells in both the undifferentiated and differentiated conditions, indicating that deletion of *Cep215* does not affect proliferation of P19 cells (Fig. 2i, Supplementary Fig. 2).

**Figure 3:**
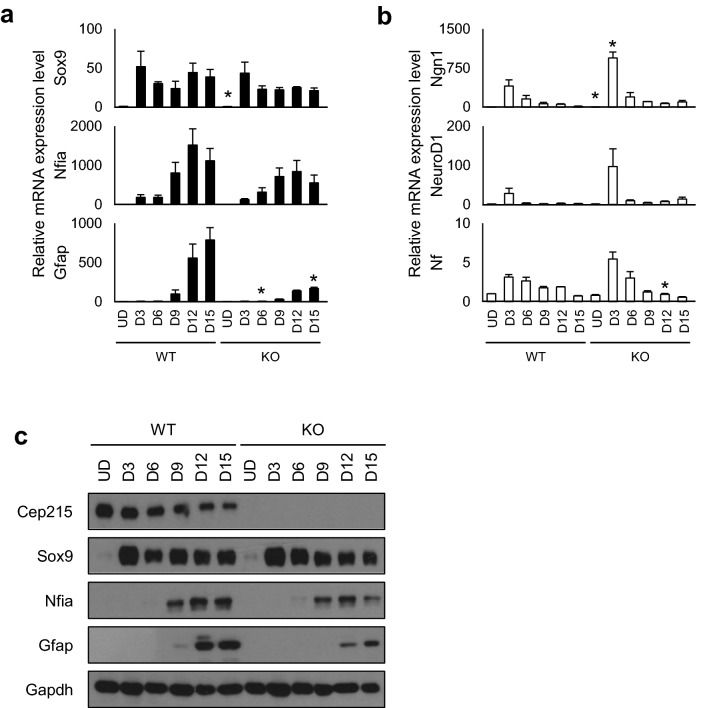
Expression of the cell fate determinants in *Cep215*-deleted P19 cells. The wild type (WT) and *Cep215*-deleted (KO) P19 cells were differentiated up to D15. (**a**, **b**) The cells at indicated days of differentiation were subjected to qRT-PCR analysis with indicated glial cell fate (**a**) and neuronal cell fate (**b**) determinant genes. Relative mRNA levels of each factor were normalized to the Gapdh levels, and compared to the undifferentiated (UD) WT levels. (**c**) The protein levels of the glial cell fate determinants were analyzed with immunoblot using antibodies specific to Cep215, Sox9, Nfia, Gfap, and Gapdh. (**a**, **b**) The statistical analysis was performed by unpaired *t*-test. Error bars, SEM. *P < 0.05.

Cell fate is determined by expression of a specific set of genes at early stages. For glial cell determination, Sox9 is one of the initiators and Nfia is one of the downstream determinants^24–26^. For neuronal cell differentiation, Ngn1 and NeuroD1 are known as cell fate initiators^27,28^. We observed expression of selected cell fate determinants in P19 cells undergoing differentiation. Quantitative reverse transcription PCR (qRT-PCR) analysis revealed that *Sox9* expression was immediately induced and *Nfia* expression followed in both wild type and *Cep215*-deleted P19 cells (Fig. 3a). Similar expression kinetics were also observed at the protein levels (Fig. 3c). However, expression of Gfap, a structural protein for astrocytes, appeared reduced in *Cep215*-deleted cells at both the mRNA and protein levels (Fig. 3a,c). These results indicate that Cep215 is not involved in cell fate determination toward astrocytes but is necessary for expression of Gfap for morphological differentiation. We also determined expression of neuronal cell determinants, Ngn1 and NeuroD1, in P19 cells. The results showed that both *Ngn1* and *NeuroD1* were immediately expressed after RA treatment (Fig. 3b). We did not observe a significant difference in their expression and Nf-L, a structural protein for neuronal cells, in wild type and *Cep215*-deleted cells, indicating that Cep215 is not involved in cell fate determination and differentiation of neuronal cells (Fig. 3b).

### Reduction of the microtubule organizing activity in *Cep215*-deleted P19 cells

**Figure 4:**
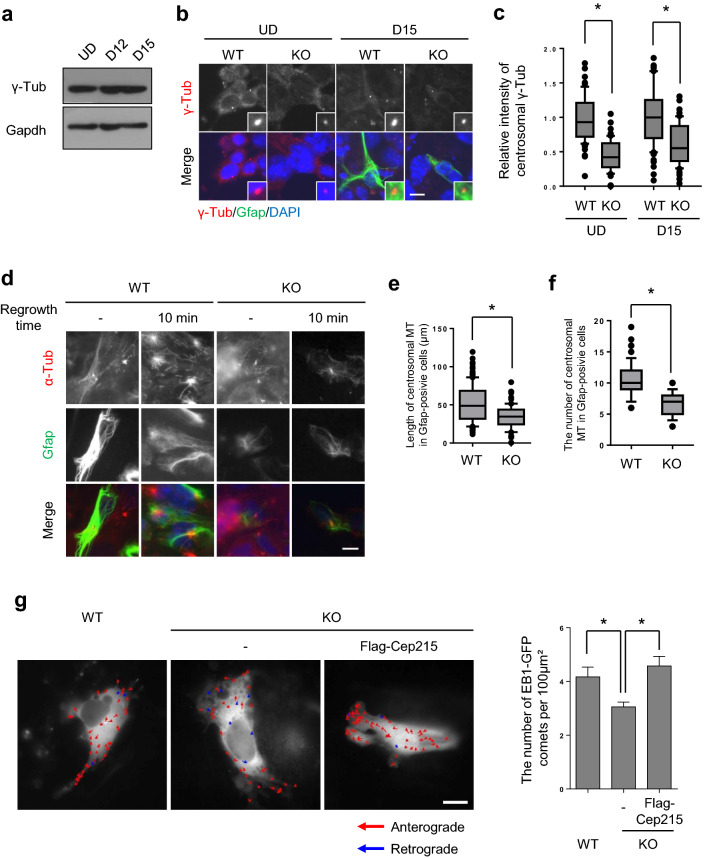
Reduction of the microtubule organizing activity in *Cep215*-deleted P19 cells. (**a**, **b**) Undifferentiated (UD) and differentiated (D12 and D15) P19 cells were subjected to immunoblot (**a**) and coimmunostaining (**b**) analyses with antibodies specific to γ-tubulin, Gapdh and Gfap. (**c**) Intensities of the centrosomal γ-tubulin signals of undifferentiated and differentiated cells were measured. In case of differentiated cells, only Gfap-positive cells were analyzed. (**d–f**) *Cep215*-deleted P19 cells at D12 were treated with nocodazole for 1 h and transferred to a fresh medium for microtubule regrowth. Ten minutes later, the cells were subjected to coimmunostaining analysis with antibodies specific to α-tubulin (red) and Gfap (green). Total length of centrosomal microtubules (**e**) and the number of centrosomal microtubules (**f**) were determined. (**g**) EB1-GFP was transfected into *Cep215*-deleted P19 cells with and without ectopic Flag-Cep215. The movements of EB1 comets were traced and indicated as red and blue arrows for anterograde and retrograde directions, respectively. Real-time movements of the EB1 comets were shown in Supplementary Movie 1. The number of EB1-GFP signals was counted and normalized with cell area. Twenty cells per experimental group were measured in three independent experiments. (**b**, **d**) Nuclei were stained with DAPI (blue). (**b**, **d**, **g**) Scale bar, 10 μm. (**c**, **e**, **f**) Greater than 60 cells per experimental group were measured in three independent experiments. (**c**, **e**, **f**, **g**) The statistical significance was determined by two-way ANOVA (**c**), unpaired *t*-test (**e**, **f**) or one-way ANOVA (**g**). Error bars, SEM. *P < 0.05.

We investigated centrosome functions in the *Cep215*-deleted P19 cells. First, immunoblot and immunostaining analyses were performed to determine the expression of γ-tubulin in differentiated P19 cells. The results showed that the total amount and centrosomal levels of γ-tubulin remained constant during glial differentiation (Fig. 4a–c). However, the centrosomal levels of γ-tubulin were significantly reduced in *Cep215*-deleted cells (Fig. 4b,c). These results are consistent with previous report that Cep215 plays a role in recruitment of γ-tubulin into the centrosomes^7^.

We determined the microtubule organizing activity in *Cep215*-deleted P19 cells. The differentiated P19 cells were treated with nocodazole for 1 h and transferred into a fresh medium for microtubule regrowth. The results showed that the length and number of newly nucleated microtubules at the centrosome were significantly reduced in the *Cep215*-deleted astrocytes (Fig. 4d–f). We also determined microtubule formation activity in P19 cells using the EB1-GFP protein as a marker for the plus end of microtubules. We observed that the density of growing microtubules was significantly reduced in *Cep215*-deleted cells and was recovered with ectopic Flag-Cep215 (Fig. 4g, Supplementary Movie. 1a-c). These results indicate that Cep215 is essential for microtubule organization in differentiated astrocytes.

### Importance of interaction between Cep215 and γ-TuRC for glial differentiation

**Figure 5:**
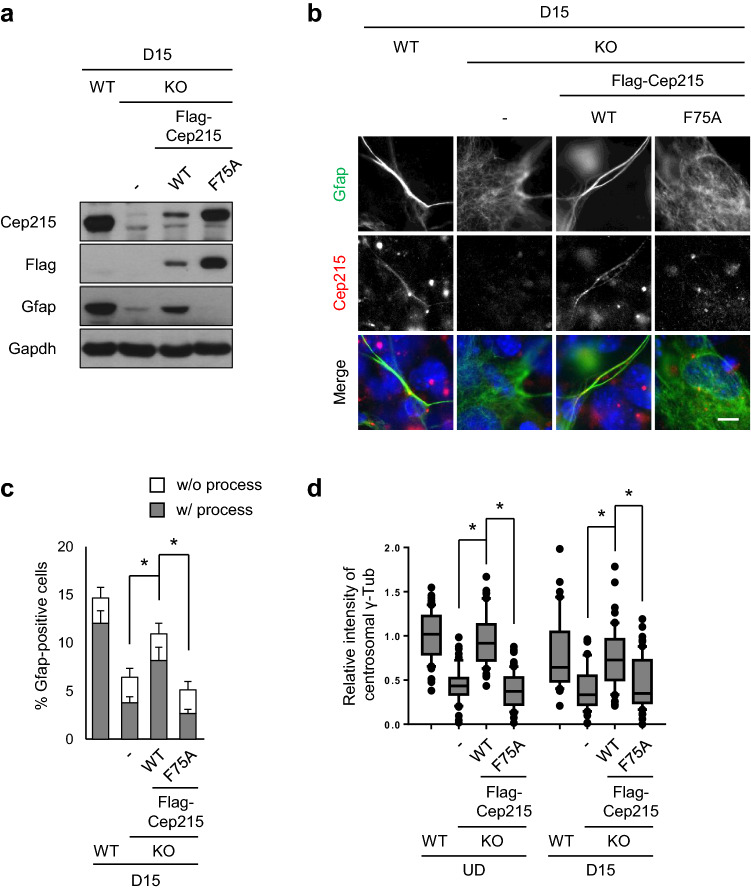
Importance of Cep215 interaction with γ-TuRC in glial differentiation. *Cep215*-deleted P19 cells were stably transfected with Flag-Cep215 (WT) and Flag-Cep215^F75A^ (F75A). (**a**, **b**) The cells at D15 were subjected to immunoblot (**a**) and coimmunostaining (**b**) analyses with antibodies specific to Cep215, Flag, Gfap and Gapdh. Nuclei were stained with DAPI (blue). Scale bar, 10 μm. (**c**) The number of Gfap-positive cells with or without glial processes were counted. Greater than 1400 cells per experimental group were counted in three independent experiments. (**d**) Intensities of the centrosomal γ-tubulin signals in undifferentiated (UD) and differentiated (D15) cells were measured. In case of cells at D15, only Gfap-positive cells were subjected to analysis. Greater than 60 cells per experimental group were analyzed in three independent experiments. (**c**, **d**) The statistical analysis was performed by two-way ANOVA. Error bars, SEM. *P < 0.05.

We examined importance of the Cep215 function as a recruiter of the γ-tubulin ring complex (γ-TuRC) during astrocyte differentiation. A point mutant of Cep215 at phenylalanine 75 position (Cep215^F75A^) is reported to have a defect in interaction with γ-TuRC^29^. We rescued the knockout P19 cells with the *Cep215* mutant construct and determined astrocyte differentiation. Immunoblot analysis confirmed that comparable amounts of ectopic Cep215 proteins were expressed (Fig. 5a). The results also showed that the cellular levels of Gfap were rescued with Flag-Cep215, but not with Flag-Cep215^F75A^ (Fig. 5a). Furthermore, the ectopic Cep215^F75A^ hardly rescued the glial process formation activity (Fig. 5b,c). These results suggest that Cep215 interaction with γ-TuRC is critical for astrocyte differentiation.

We determined the centrosomal levels of γ-tubulin in the Cep215^F75A^–rescued P19 cells. The results showed that the centrosomal γ-tubulin levels were reduced in the *Cep215*-deleted P19 cell (Fig. 5d). Ectopic expression of wild type Cep215 rescued the phenotypes, but that of Cep215^F75A^ did not (Fig. 5d). These results emphasize the importance of Cep215 function in recruitment of γ-TuRC in astrocyte differentiation.

### Subcellular localization of Cep215 in primary cultured astrocytes

**Figure 6:**
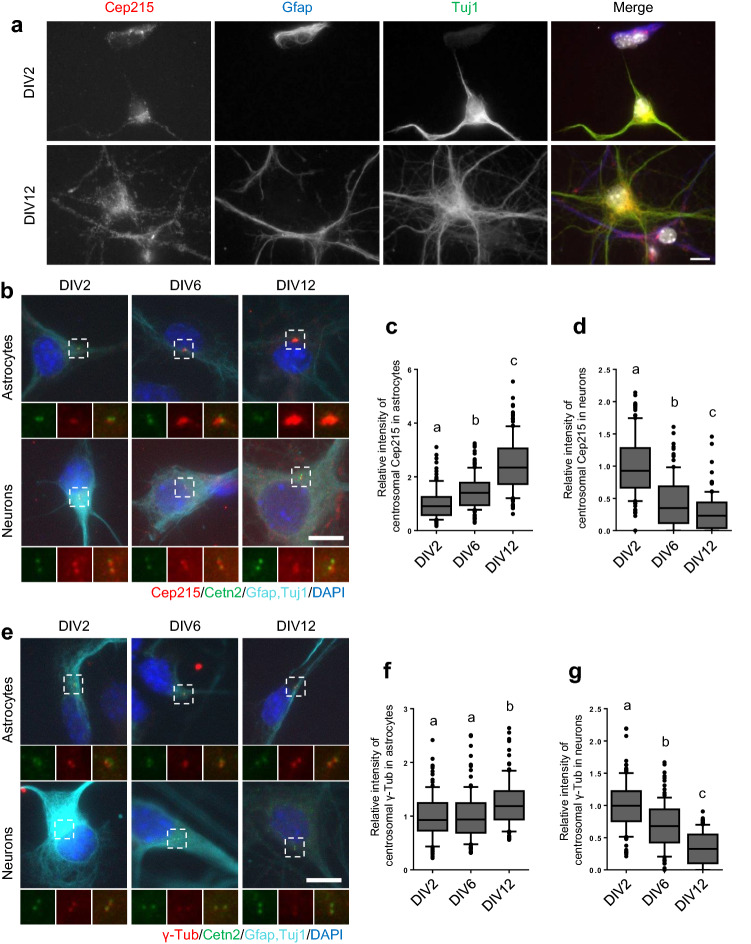
Subcellular distribution of Cep215 in mouse hippocampal cells. (**a**) Mouse hippocampal cells were cultured to early (DIV2) and late (DIV12) developmental stages. The cells were coimmunostained with antibodies specific to Cep215 (red), Gfap (blue) and Tuj1 (green). Nuclei were stained with DAPI (white). (**b**–**d**) Cultured mouse hippocampal cells at DIV2, 6 and 12 were coimmunostained with antibodies specific to Cep215 (red) and centrin-2 (green) along with cell markers (Gfap or Tuj1, cyan). Intensities of the centrosomal Cep215 signals were measured in astrocytes (**c**) and neurons (**d**). (**e**–**g**) Cultured hippocampal cells at DIV2, 6 and 12 were subjected to coimmunostaining analysis with antibodies specific to γ-tubulin (red) and centrin-2 (green) along with cell markers (Gfap or Tuj1, cyan). Intensities of the centrosomal γ-tubulin signals were measured in astrocytes (**f**) and neurons (**g**). (**b**, **e**) Nuclei were stained with DAPI (blue). (**a**, **b**, **e**) Scale bar, 10 μm. (**c**, **d**, **f**, **g**) Greater than 90 cells per experimental group were measured in three independent experiments. The statistical significance was analyzed using one-way ANOVA and is indicated by lower cases (*P* < 0.05).

Our results with P19 cells revealed that Cep215 is required for morphological differentiation of astrocytes. We wished to confirm our observations in primary culture cells from mouse embryonic hippocampus. First, we determined subcellular distribution of Cep215 in the hippocampal cells. The immunostaining analysis revealed that Cep215 signals were not limited to the centrosomes but were also detected at the cytoplasm of cultured cells (Fig. 6a). Cep215 signals were coimmunostained with Gfap, which is consistent with the previous observations in differentiated P19 cells (Fig. 6a). We also observed Cep215 signals in the cell body of Tuj1-positive cells, suggesting that Cep215 might have a role in cytoplasmic microtubule organization in neurons (Fig. 6a). We determined the centrosomal levels of Cep215 during a prolonged culture and observed that the centrosomal levels of Cep215 increased in astrocytes whereas the opposite was the case in neurons (Fig. 6b–d). Similar expression patterns were observed in the centrosomal levels of γ-tubulin (Fig. 6e–g). These results suggest that Cep215 may differentially function during morphogenesis in astrocytes and neurons.

### Depletion of Cep215 in mouse hippocampal cells

**Figure 7:**
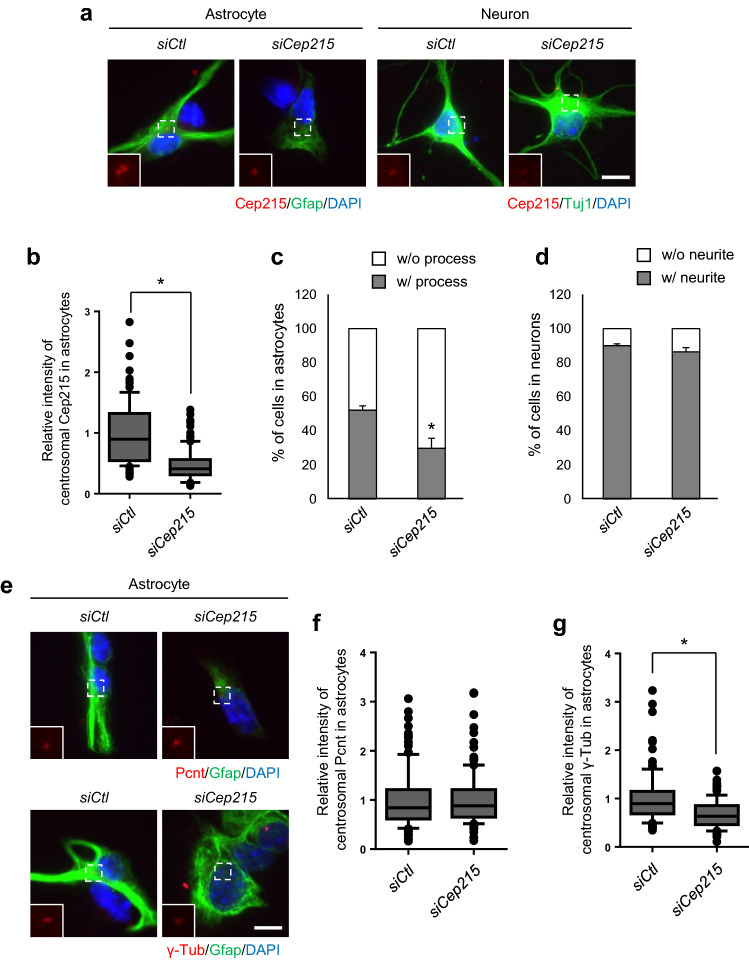
Involvement of Cep215 in morphological differentiation of hippocampal astrocytes. Cep215 in primary hippocampal cells was depleted with specific siRNA (*siCep215*) transfection at DIV1. (**a–d**) The cells at DIV3 were subjected to coimmunostaining analysis with antibodies specific to Cep215 (red) along with Gfap or Tuj1 (green). (**b**) Intensities of the centrosomal Cep215 signals in astrocytes were measured. The number of Gfap-positive (**c**) and Tuj1-positive (**d**) cells with and without processes was counted. (**e**–**g**) The Cep215-depleted hippocampal cells at DIV3 were subjected to coimmunostaining analysis with pericentrin (Pcnt) or γ-tubulin (red) along with Gfap (green). Intensities of the centrosomal Pcnt (**f**) and γ-tubulin (**g**) signals were measured in Cep215-depleted astrocytes. (**a**, **e**) Nuclei were stained with DAPI (blue). Scale bar, 10 μm. (**b**, **f**, **g**) Greater than 90 cells per experimental group were estimated in three independent experiments. (**c**, **d**) More than 420 cells per experimental group were counted in three independent experiments. (**b**, **c**, **d**, **f**, **g**) The statistical significance was determined by unpaired *t*-test. Error bars, SEM. *P < 0.05.

To test the hypothesis in which Cep215 may be involved in astrocyte morphogenesis, we transfected siRNA specific to Cep215 (*siCep215*) into the primary hippocampal cells. Depletion of Cep215 in astrocytes and neurons was confirmed by immunostaining analyses of the centrosomes (Fig. 7a,b). At the same time, we observed that the number of astrocytes without glial processes increased after depletion of Cep215 (Fig. 7c). On the other hand, Cep215 depletion did not affect neurite formation (Fig. 7d).

We determined the centrosomal levels of pericentrin and γ-tubulin in Cep215-depleted astrocytes (Fig. 7e). The results showed that the centrosomal levels of γ-tubulin were reduced in Cep215-depleted astrocytes, whereas those of pericentrin were not (Fig. 7f,g). These results suggest that depletion of Cep215 might cause reduction of the microtubule organizing activity of the centrosomes in astrocytes, which results in defects in process formation.

## Discussion

In this work, we investigated involvement of Cep215 in morphological differentiation of astrocytes. Cep215 localized at the glial processes as well as at the centrosomes during astrocyte differentiation. Furthermore, the formation of glial processes was suppressed in the *Cep215*-deleted P19 cells. Similar phenotypes were also observed in astrocytes in the embryonic hippocampal culture. Our results indicate that Cep215 is essential for glial process formation during differentiation of astrocytes.

The major biological function of Cep215 is to recruit γ-TuRC at specific sites of the cell for microtubule organization^9^. In fact, we observed a significant reduction of the microtubule organization activity in the *Cep215*-deleted P19 cells even after astrocyte differentiation (Fig. 4). The microtubule organizing function of Cep215 should be critical for the glial process formation, since the ectopic Cep215^F75A^ protein could not rescue the *Cep215* deletion phenotypes in astrocyte differentiation of P19 cells (Fig. 5). Since the cellular distribution of Cep215 is not limited to the centrosome, it is likely that the deletion phenotypes of *Cep215* are partly attributed by cytoplasmic Cep215. It was already known that Cep215 controls the cytoplasmic microtubule organization^29^. Likewise, the regrowth of cytoplasmic microtubule should be reduced in *Cep215*-deleted astrocytes compared to wild type P19 cells (Fig. 4d). These results imply that Cep215 may regulate the organization of microtubule in astrocytes in both the centrosome and cytoplasm. Further experiments are needed to clarify the role of non-centrosomal Cep215 in glial differentiation.

Importance of cytoplasmic microtubule organization in cell morphogenesis has been demonstrated in number of tissues^30^. For example, the cytoplasmic γ-tubulin in mature neurons increases to maintain the microtubule nucleating activity in the axon^23^. We propose that the cytoplasmic microtubule organization is also essential for glial process formation of astrocytes. It is possible that Cep215 anchors γ-TuRC at the glial processes using a specific subcellular organelle whose identity remains to be discovered. However, we do not rule out the possibility that microtubules may be nucleated at the centrosome and transported to the intended location through existing microtubule networks^31^. In accord, the centrosomal levels of Cep215 significantly increased in mouse hippocampal culture (Fig. 6), and microtubule organizing activity of centrosome was still functional for microtubule organization during astrocyte development (Fig. 4).

Specific roles of microtubules in glial process formation have not been fully understood yet. Microtubules may extend the cell periphery for glial process formation. Microtubules, then, provide an intracellular transport route for glial process formation. For example, mitochondria should be distributed along the glial processes. In fact, mitochondria are aligned with and transported along microtubules in cultured astrocytes^32^. Gfap is also known to be transported through microtubules when astrocytes start to extend the glial processes^21,33^. We observed that *Cep215* deletion suppressed the cellular Gfap levels (Fig. 2). We suspect that *Gfap* expression may be stimulated when cell periphery is extended by microtubule networks. Both *Gfap* mRNA and protein could be transported through microtubules to install glial processes during gliogenesis^34,35^.

*Cep215* is one of causal genes of microcephaly^36^. A mouse genetic study revealed that Cep215 is essential for proliferation and differentiation of neurons during brain development^16^. Nonetheless, it remains elusive how microcephaly is developed by *Cep215* mutations. It was initially proposed that defects in spindle pole orientation reduce population of neuronal stem cells during brain development^37^. It was later proposed that extensive cell death might cause reduction of brain cell number during development^38^. In *Drosophila*, Cnn is known to be important for dendrite morphogenesis^19^. Therefore, Cep215 is likely to be involved in multiple stages of neuronal differentiation during brain development.

Our work proposes another possibility that defects in glial differentiation might contribute to microcephaly development in *Cep215* mutant patients. Astrocytes are the most abundant cell types in the central nervous system with a remarkable heterogeneity both in morphology and function. In the past, astrocytes were believed to act as passive supporting cells for neuronal connections and tissue homeostasis. However, recent works revealed that astrocytes play diverse roles in the brain, such as the formation of neural networks, neurotransmitter recovery, extracellular ion maintenance, and detoxification^39^. Morphology of glial processes is very diverse depending on where they reside in the brain. The positioning, size, and anatomical complexity of protoplasmic astrocytes make these cells well-suited to interaction with neurons and synaptic elements in the brain. In fact, defects in astrocyte development are known to link to neurological disease including diverse neurodegenerative diseases and neurodevelopmental disorders^39^. In the future, it is worth to investigate specific functions of Cep215 in astrocytes in vivo.

## Materials and methods

### Cell culture and induction of differentiation

Cell culture and differentiation method were performed following Yoon et al.^40^ with minor modifications. The P19 mouse embryonic carcinoma cells were maintained in DMEM (Welgene) supplemented with 10% FBS (Welgene) and antibiotics (ANT-MPT; Invivogen) at 37 °C. To induce differentiation of P19 cells, 1.0 × 10^5^ cells/ml were suspension cultured in 100 mm bacterial petri dishes, and treated with 1 μM all trans-retinoic acid (R2656; Sigma) once a day for 4 days. Four days later, the aggregated embryoid bodies were dissociated with pre-warmed 0.05% trypsin-0.53 mM EDTA (Welgene), and replated on the poly-L-lysine (P4707; Sigma) coated dishes with 3.2 × 10^2^ cells/ml. In case of the microtubule regrowth assays and EB1-GFP comet assays, 2.4 × 10^5^ cells/ml were seeded on the coated dishes to prevent the cells from peeling off. The seeded cells were maintained in DMEM supplemented with 10% FBS for glial differentiation. In case of neuronal differentiation, the dissociated embryoid bodies were seeded on poly-L-lysine/Laminin double-coated dishes with 1.2 × 10^4^ cells/ml. The cells were maintained in DMEM supplemented with B27 supplement. To estimate the Gfap- or Tuj1- positive population, the number of Gfap or Tuj1 expressing cells were counted among all nuclei in several microscopic fields. And then, Cells with mesh-like structures of Gfap or Tuj1 around the cell body were counted as cells without processes. Cells with long and thick processes and protrusions from their cell body were counted as cells with processes.

Mouse hippocampal cultures were prepared from E18 mouse embryos of either sex as modified protocols from Beaudoin et al.^41^. Dishes and coverslips were coated with 0.1 mg/ml poly-D-lysine solution diluted by borate buffer. Dissociated hippocampi tissues were treated with papain (1.2 mg/ml) for 20 min at 37 °C. The tissues were then mechanically dissociated by titration with a micropipette tip. Hippocampal cells 4 or 8 × 10^4^ cells/ml were plated in MEM (Welgene) supplemented with 0.6% glucose (Sigma), 1 mM sodium pyruvate (Sigma), antibiotics (ANT-MPT; Invivogen), 2 mM L-glutamine (Welgene) and 10% FBS (Welgene) for 4–5 h before exchange with a neurobasal medium (Gibco) containing 0.5 mM L-glutamine and B27 supplement (Gibco). The cells were maintained in a 5% CO_2_ incubator at 37 °C for up to 12 days. At DIV4 and DIV11, a half of the original media was discarded and replaced with fresh neurobasal media supplemented with 0.5 mM glutamine and B27 supplement.

### Generation of *Cep215* knockout cells and stable cell lines

RNA-guided targeting of mouse *Cep215* in P19 cells was achieved through transfection of the *Cas9* vector including guide RNA (gRNA). The *Cas9* vector *pSpCAS9(BB)-2A-Puro* was purchased from Addgene, and gRNA was subcloned as described^42^. The 23 base pair genomic targeting sequence of the mouse *Cep215* gRNA was 5′-AAGAGGAAGGAAGGCGCCCTGCC-3′. P19 cells were transfected with 2 μg of the *Cas9* vector containing gRNA using Lipofectamine 3000 (Invitrogen), and selected using 1.5 μg/ml of puromycin (Calbiochem) for 2 days, and then puromycin was washed with fresh media. The in-del mutation was detected about 87% of cells using the surveyor mutation detection kit (Transgenomic), these cells were seeded by single cell in 96-well plates for generation of monoclonal cell line. The used monoclonal *Cep215*-deleted cell was confirmed that the endogenous Cep215 is not detected using both immunoblot and immunocytochemistry. Finally, to determine *Cep215* deletion, the genomic amplicons targeted by Cas9 nuclease were extracted from previous monoclonal cells, and cloned into a plasmid. Two types of deletion were confirmed from all tested amplicons (over 20) using Sanger sequencing.

To generate P19 cell lines stably expressing the *Flag-Cep215* constructs, the plasmid DNA was transfected in P19 cells using Lipofectamine 3000 (Invitrogen) according to the manufacturer’s instruction. The P19 cell and *Cep215*-deleted cell lines stably expressing *Flag-Cep215* constructs were generated using 800 μg/ml of G418 (Calbiochem) selection.

### Plasmids and RNA interference

The wild type and F75A mutant *Cep215* cDNA were initially subcloned into *pCMV10-3* × *Flag* vector. Then, the *CMV* promoter was replaced to *SV40* promoter (*pSV40-3* × *Flag*) to regulate the expression level of ectopic proteins as much as endogenous Cep215. The *EB1* cDNA was subcloned into *pEGFP-N1* vector. The siRNA specific to mouse *Cep215* (5′-CUCAGUGCAGUGAGGCUAUUATT-3′) was purchased from ST Pharm, and was transfected using RNAiMAX (Invitrogen) according to the manufacturer’s instruction. Non-specific control siRNA (5′-GCAATCGAAGCTCGGCTAC-3′) was used.

### Reverse Transcription and quantitative real-time RT-PCR (qRT-PCR)

qRT-PCR analysis was performed following Yoon et al.^43^ with minor modifications. Total RNA was isolated from undifferentiated or differentiated P19 cells at the indicated days using TRIzol reagent (Invitrogen) following the protocol of the manufacturer. Reverse transcription was conducted with random hexamers. Real-time PCR was carried out with the Applied Biosystems (Carlsbad, CA) 7300 Real Time PCR System. The primer sequences are listed in Table 1. Real-time PCR was conducted in triplicate, and the experiment was repeated three times. Relative mRNA expression level was determined with the comparative Ct method and normalized to endogenous Gapdh.

**Table 1:** Primer sequences for qRT-PCR.

Gene	Sequence	References
Gfap	F: 5′-AAG CCA AGC ACG AAG CTA ACG A-3′ R: 5′-TTG AGG CTT TGG CCC TCC-3′	^44^
Sox9	F: 5′-AGG AAG CTG GCA GAC CAG TA-3′ R: 5′-TCC ACG AAG GGT CTC TTC TC-3′	^45^
Nfia	F: 5′-GGC ATA CTT TGT ACA TGC AGC-3′ R: 5′-ACC TGA TGT GAC AAA GCT GTC C-3′	^46^
Neurofilament	F: 5′-TGA TGT CTG CTC GCT CTT TC-3′ R: 5′-CTC AGC TTT CGT AGC CTC AAT-3′	^47^
Ngn1	F: 5′-GAC ACT GAG TCC TGG GGT TC-3′ R: 5′-GTC GTG TGG AGC AGG TCT TT-3′	^48^
NeuroD1	F: 5′-GGA GGA GGA GGA TCA AAA GC-3′ R: 5′-TGG GTC TTG GAG TAG CAA GG-3′	^48^
Gapdh	F: 5′-TCA AGA AGG TGG TGA AGC AG-3′ R: 5′-AGG TGG AAG AGT GGG AGT TG-3′	^43^

### Immunoblot analyses

Protein sampling and immunoblot analysis were performed following Shin et al.^49^ with minor modifications. In brief, the undifferentiated and differentiated P19 cells were lysed using RIPA buffer (150 mM NaCl, 1% Triton X-100, 0.5% sodium deoxycholate, 0.1% SDS, 50 mM Tris–HCl at pH 8.0, 10 mM NaF, 1 mM Na_3_VO_4_, 1 mM EDTA and 1 mM EGTA) containing a protease inhibitor cocktail (P8340; Sigma-Aldrich). Cell lysates were centrifuged with 12,000 rpm for 10 min at 4℃. The supernatants were mixed with 4 × SDS sample buffer (250 mM Tris–HCl at pH 6.8, 8% SDS, 40% glycerol and 0.04% bromophenol blue) and 400 mM DTT (0,281-25G; Amresco). Mixtures were boiled for 5 min and the protein samples were subjected to SDS–polyacrylamide gel electrophoresis. The gel was transferred to a nitrocellulose membrane. The membrane was blocked with 5% skim milk in 0.1% TBST (Tris-buffered saline TBS with 0.1% Tween 20) for 1–2 h. The membrane was incubated with primary antibody overnight at 4˚C. The membrane was incubated with horseradish peroxidase-conjugated secondary antibody for 30–40 min at room temperature. The ECL solution was treated onto the membrane, and then exposed to an X-ray film.

### Immunostaining and image processing

Immunostaining analysis was performed following Shin et al.^49^ with minor modifications. In brief, the cells on the coverslip were fixed with cold methanol for 10 min on ice and washed with phosphate-buffered saline (PBS) 3 times. The fixed cells were blocked with 3% bovine serum albumin in 0.5% PBST (PBS with 0.5% Triton X-100) for 30 min, incubated with the primary antibody for 1 h. After washing with 0.1% PBST (PBS with 0.1% Triton X-100) 3 times, the cells were incubated with the secondary antibody for 30 min, and then washed with 0.1% PBST 3 times. The coverslips were mounted on a slide glass using Prolong gold mounting solution (Invitrogen) after DAPI incubation.

In case of triple staining, the permeabilization step is necessary. For permeabilization, the cells were fixed and incubated with 0.1% PBST for 10 min. The cells were incubated with primary (rabbit Cep215 or rabbit γ-Tub antibodies and mouse Tuj1 or mouse Cetn2 antibodies) and secondary (anti-rabbit-488 nm- and anti-mouse-647 nm-fluorescent-conjugated) antibodies as same with method mentioned above. Then, the third (mouse Tuj1 and mouse Gfap) antibodies were prepared with Zenon Alexa fluor labeling kit (Z25005, Z25105; Invitrogen) following the protocol of the manufacturer. The cells were incubated with conjugated antibody for 2–3 h. The cells were incubated with DAPI and mounted on a slide glass.

The immunostained cells were observed using fluorescence microscope with a CCD (Qi-cam Fast 1394; Qimaging) camera and processed with ImagePro 5.0 (Media Cybernetics, Inc.), Adobe Photoshop software and ImageJ software. The intensity of centrosomal proteins levels was measured using ImageJ software by drawing the circle including the centrosome area, and background was excluded by drawing same size circle in the nearest cytoplasm.

### BrdU incorporation assay

The P19 cells were treated with BrdU (30 μM) for 24 h, fixed with cold methanol for 10 min on the ice and washed with PBS 3 times. For permeabilization, the cells were incubated with 0.1% PBST for 10 min. To denature DNA structure, the cells were incubated with pre-warmed 2 N HCl at 37 °C for 20 min and neutralized with 0.1 M borate buffer. The samples were subjected to immunostaining analysis with the BrdU antibody (B8434; Sigma).

### Antibodies

The Cep215 antibody were previously described^50^ or purchased (06-1398; Millipore). The antibodies against Tuj1 (MMS-435P-100; Covance), neurofilament 68 (13-0400; Invitrogen), Gfap (#3670; Cell Signaling), Sox9 (AB5535; Millipore), Nfia (HPA006111; Sigma), Flag (F3165; Sigma-Aldrich) and α-tubulin (ab18251; Abcam) were commercially purchased. The goat γ-tubulin (sc-7396; Santa Cruz) and rabbit γ-tubulin (ab11317; Abcam) were used for immunocytochemistry and mouse γ-tubulin (ab11316-100; Abcam) was used for immunoblot. The antibodies against centrin-2, centrobin and Gapdh were purchased from Millipore, Abcam and Ambion, respectively. The Alexa-fluorescence secondary antibodies were purchased from Invitrogen. The mouse and rabbit IgG-HRP antibodies were purchased from Sigma and Millipore, respectively.

### Microtubule regrowth assay

To depolymerize microtubules, P19 cells and *Cep215*-deleted cells in differentiation day 12 were treated nocodazole (1.65 μM) for 1 h. To induce microtubule repolymerization, the cells were incubated with pre-warmed fresh medium for 10 min, and immediately fixed with 4% paraformaldehyde in PEM buffer (80 mM PIPES pH6.9, 1 mM MgCl_2_, 5 mM EGTA, 0.5% Triton X-100) for 10 min at room temperature. The fixed cells were incubated with 0.3% PBST for 5 min to increase permeability and subjected to immunostaining. The length and the number of centrosomal microtubules were measured manually using NeuronJ software which is a sort of ImageJ plug-in.

### EB1-GFP comet assay

To acquire the time-lapse images, the P19 cells, *Cep215*-deleted cells, and Flag-Cep215-rescued cells were differentiated into glial cell in 35 mm confocal dishes. To express the EB1-GFP protein, differentiated cells were transfected using Lipofectamine 3000 (Invitrogen) according to the manufacturer’s instruction at differentiation day 11. Twenty-four hours after transfection, the cells were transferred to the chamber which is pre-warmed at 37℃ of Delta Vision (GE Healthcare Life Sciences). The fluorescence of EB1 was observed under a Delta Vision, equipped with a camera (CoolSNAP HQ, Roper Scientific) and a 60 × 1.4 NA UPlanSApo oil objective lens (Olympus). Time-lapse images were acquired every 1 s for 1 min. SoftWoRX was used to analyze the obtained images.

The microtubule density was determined by counting the number of EB1-GFP comets in entire area of the cells. The number of EB1 comets were normalized with the cell area using ImageJ software. The movements of EB1-GFP comets were tracked manually using ImageJ plugin, MTrack2. Twenty cells in each groups were measured

### Statistical analysis

For statistical analyses, experiments were independently performed three times. To calculate *P* values, all data were analyzed using the Prism 6 software (GraphPad Software) including unpaired two-tailed *t*-test, one- or two-way analysis of variant (ANOVA). In the case of ANOVA, the Tukey’s post-test was performed if *p* value is lower than 0.05.

All measured fluorescent intensities, the length and the number of microtubule were displayed with box-and-whiskers plots in Prism 6 (lines, median; vertical boxes, values from 25th and 75th; down error bars, 10th value, up error bar, 90th value; circle, outliers).

## Supplementary information

Supplementary Information.

Supplementary Information.

Supplementary Information.

Supplementary Information.
