# Supplementary material for: Cep215 is essential for morphological differentiation of astrocytes
Source: Sci Rep. 2020 Oct 12;10:17000. doi: 10.1038/s41598-020-72728-7 (PMC7550586; doi:10.1038/s41598-020-72728-7)

Title: Cep215 is essential for morphological differentiation of astrocytes

Authors: Donghee Kang[1,+], Wonjung Shin[1,+], Hyunjeong Yoo[1], Seongjae Kim[1], Seongju Lee[2] and Kunsoo Rhee[1, *]

Affiliation: [1] Department of Biological Sciences, Seoul National University, Seoul 08826, Korea.
[2]Department of Anatomy, Inha University School of Medicine, Incheon 22212, Korea

+ These authors equally contributed to this work
*Corresponding author: Kunsoo Rhee (rheek@snu.ac.kr)

Supplementary Figure 1. Neuronal differentiation was induced in wild type (WT) and *Cep215*-deleted (KO) P19 cells. Wild type (WT) and *Cep215*-deleted (KO) P19 cells were induced neuronal differentiation using neuron culture media. (**a**) The cells were immunostained with an antibody specific to Tuj1 (green). Nuclei were stained with DAPI (blue). Scale bar, 20μm. To show the pictures focused on the neuronal morphologies, Cep215 signals was omitted at the figure. (**b**) The number of Tuj1-positive cells with or without neurites were counted at indicated days of differentiation. Greater than 300 cells per experimental group were counted in three independent experiments. The statistical analysis was performed by two-way ANOVA. Error bars, SEM.

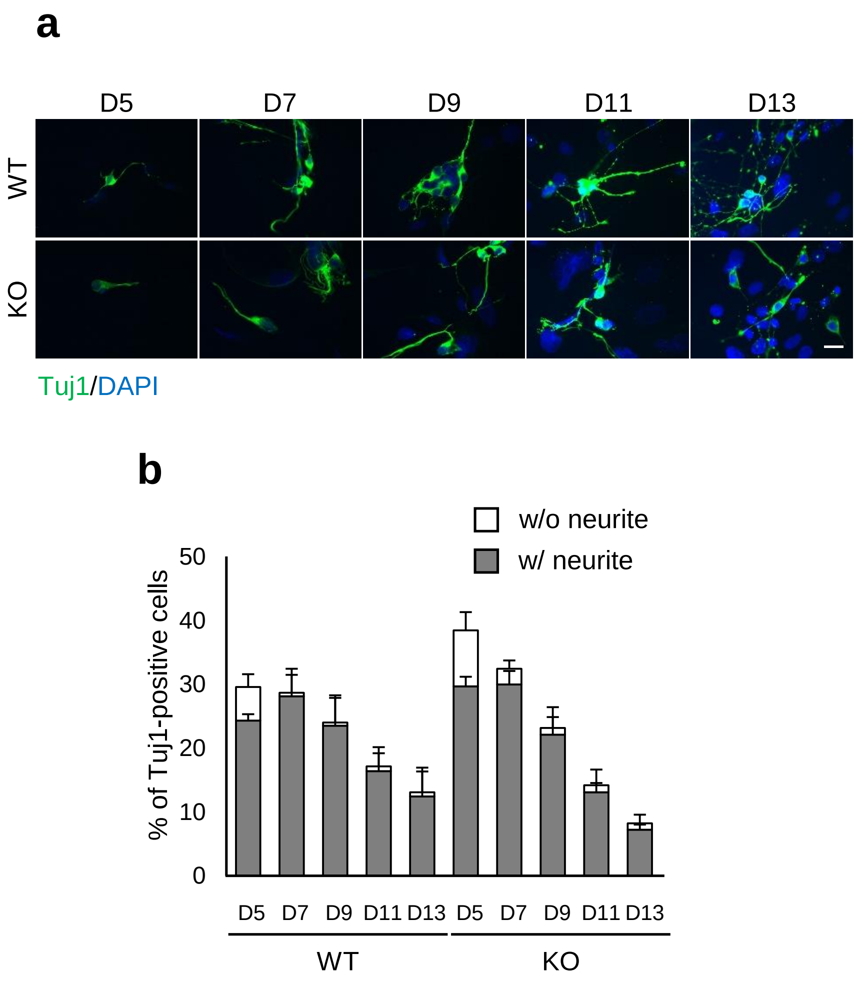

Supplementary Figure 2. BrdU incorporation assay in wild type (WT) and *Cep215*-deleted (KO) P19 cells. The undifferentiated (UD) and differentiated (D12, D15) cells were incubated with BrdU (30μM) for 24 h and were subjected to coimmunostaining analyses with antibodies specific to BrdU (green), Cep215 (red), and Gfap (yellow). Nuclei were stained with DAPI (blue). Scale bar, 20μm.

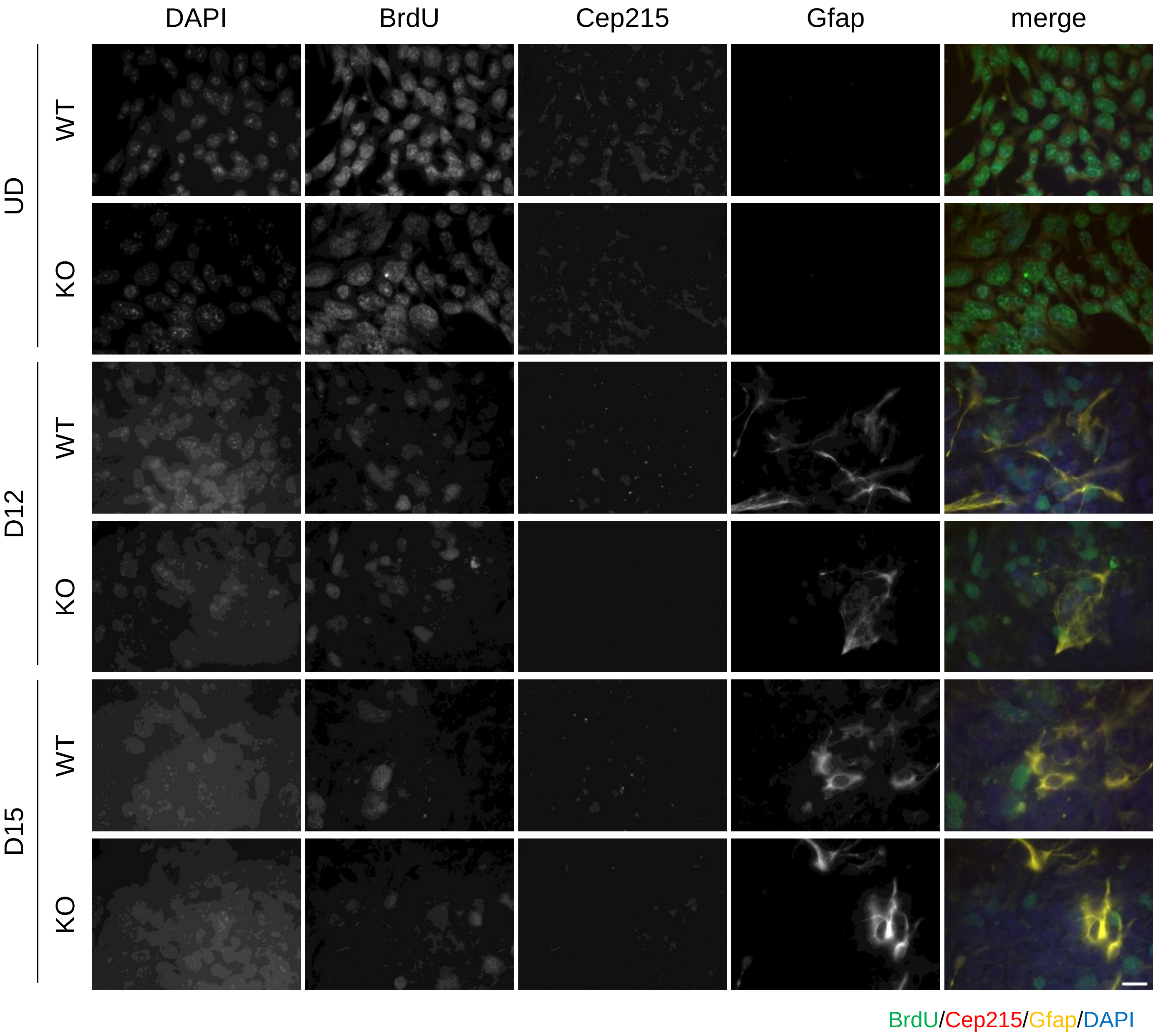

Supplementary movie 1. EB1 comet assay in *Cep215*-deleted and WT *Cep215* rescued P19 cell. Wild type (**a**), *Cep215*-deleted (**b**), and Cep215 WT rescued cells (**c**) were induced glial differentiation until D12. Before one day of the imaging, EB1-GFP construct was transfected to each cell lines. Time-lapse images were acquired every 1 second for 1 min using SoftWoRX. Sequential images were stacked to one video by using Image J.

a

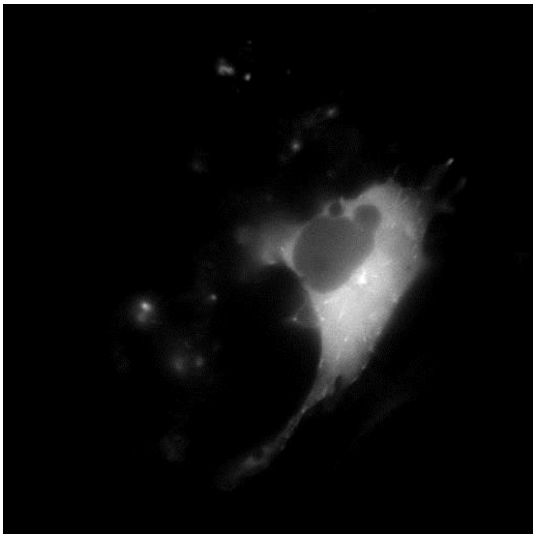

b

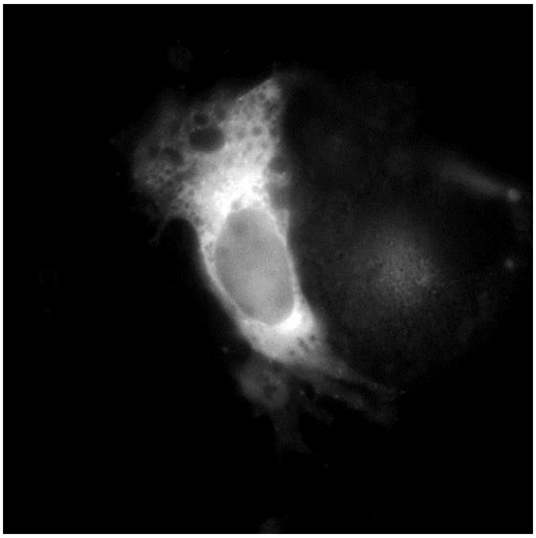

c

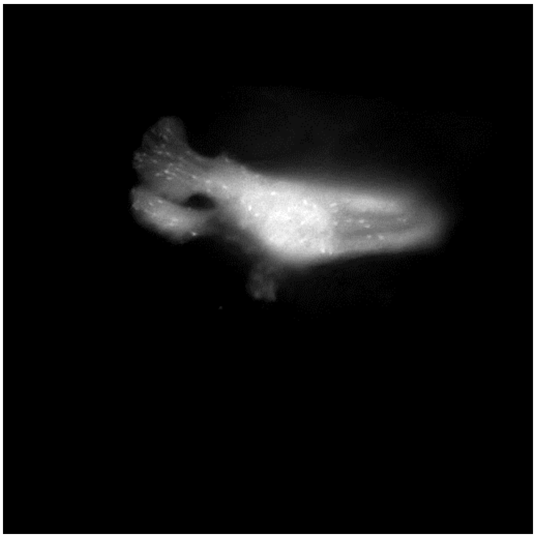

Supplementary Information 1. Full images of the immunoblots in Fig. 1b

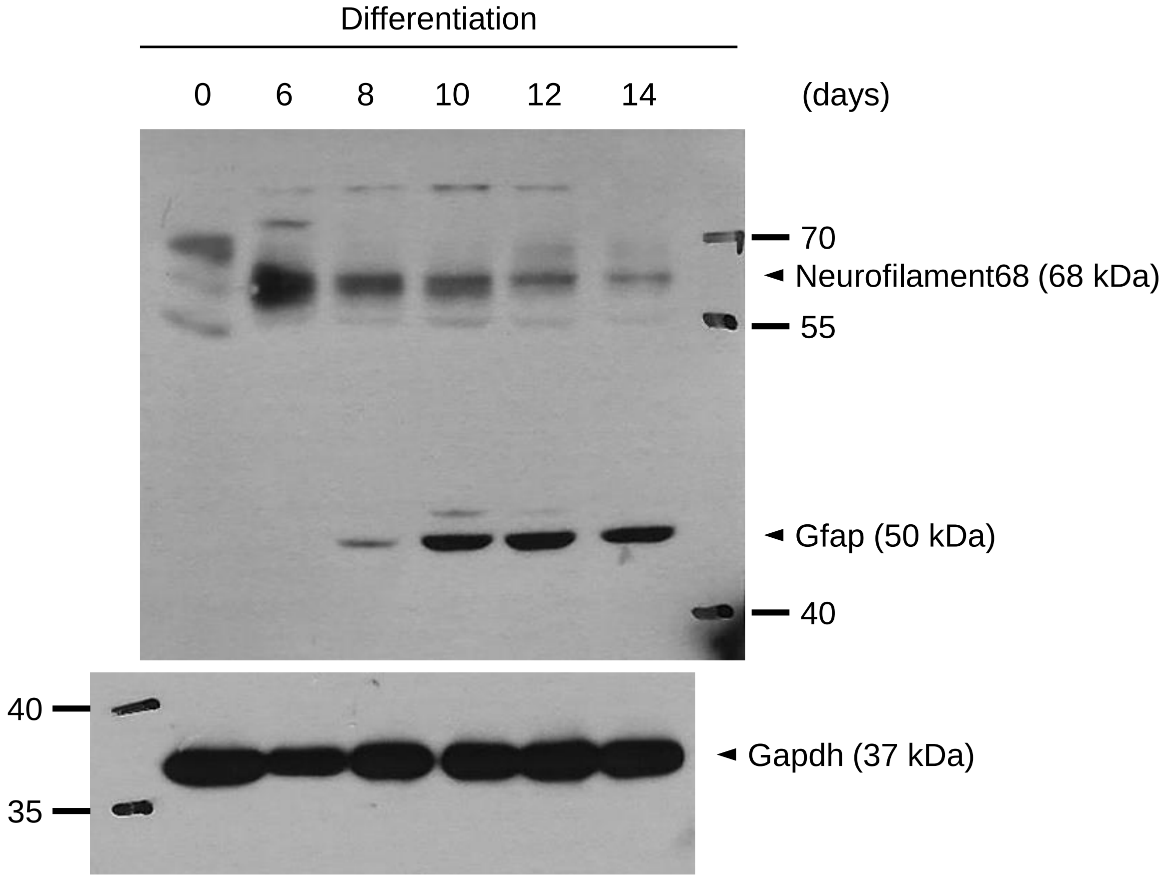

Supplementary Information 2. Full images of the immunoblots in Figs. 1d and 4a

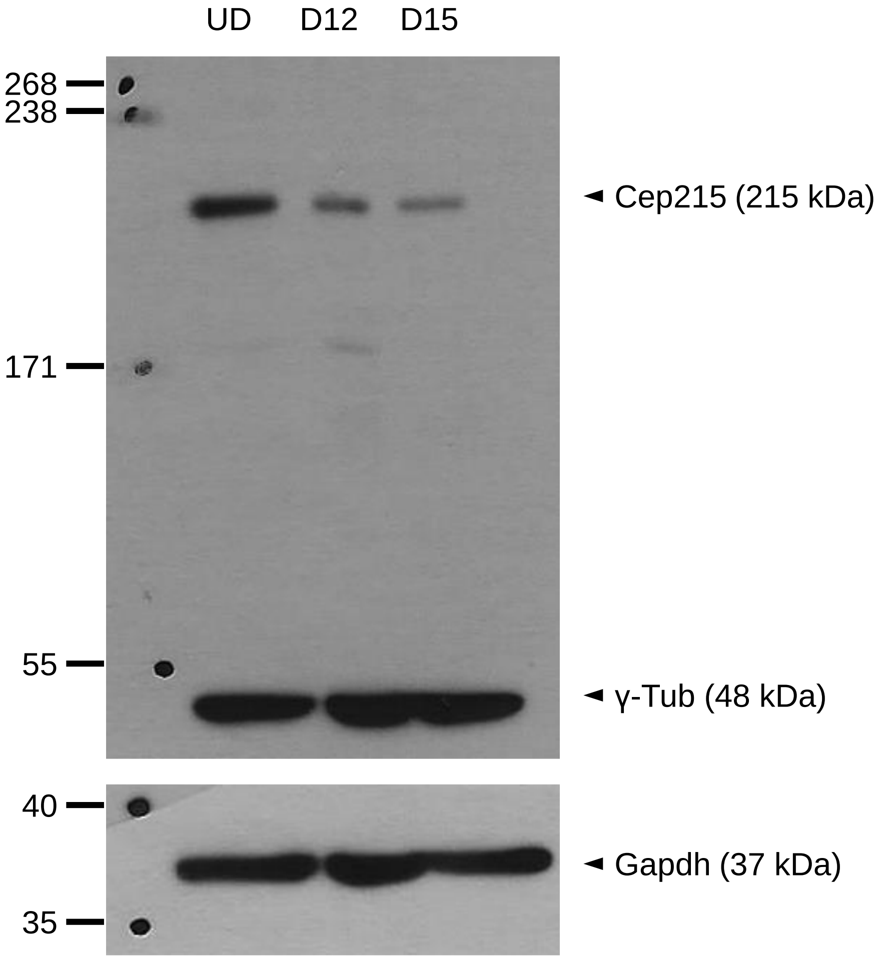

Supplementary Information 3. Full images of the immunoblots in Fig. 2a

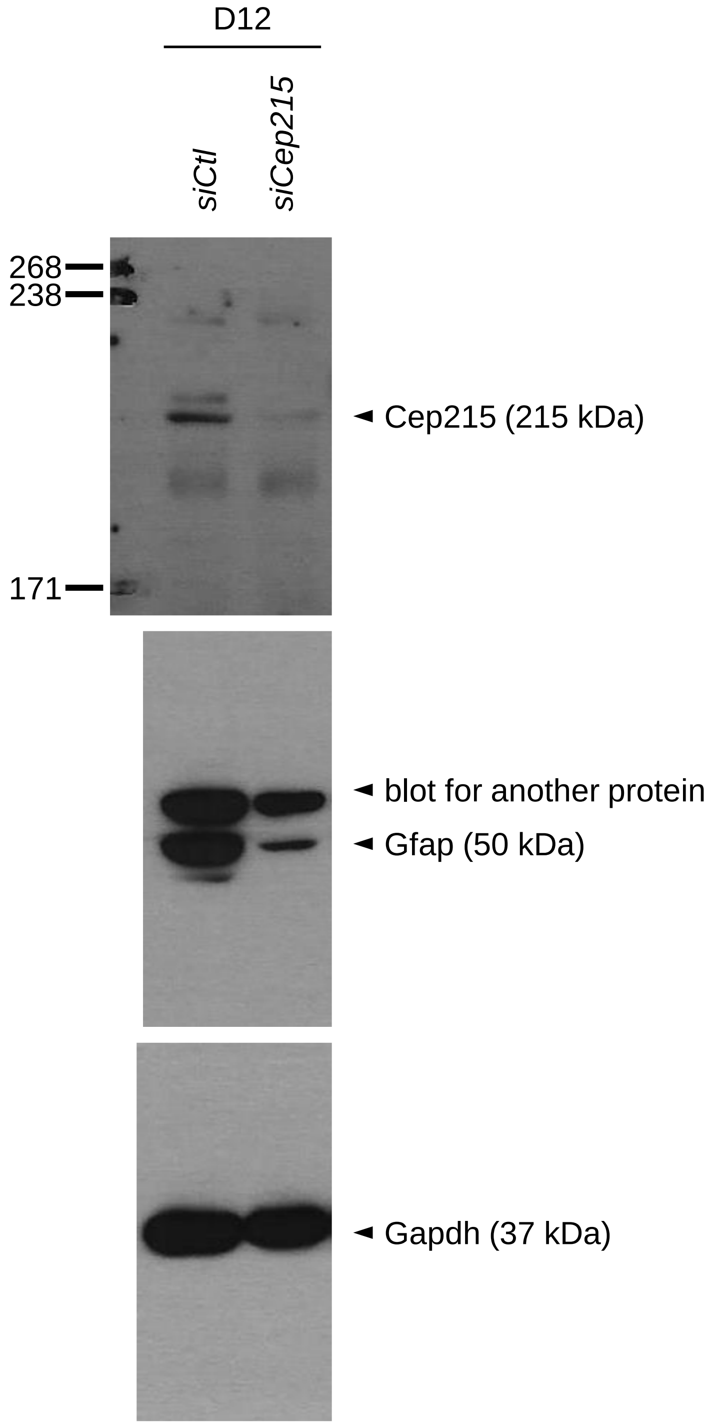

Supplementary Information 4. Full images of the immunoblots in Fig. 2d

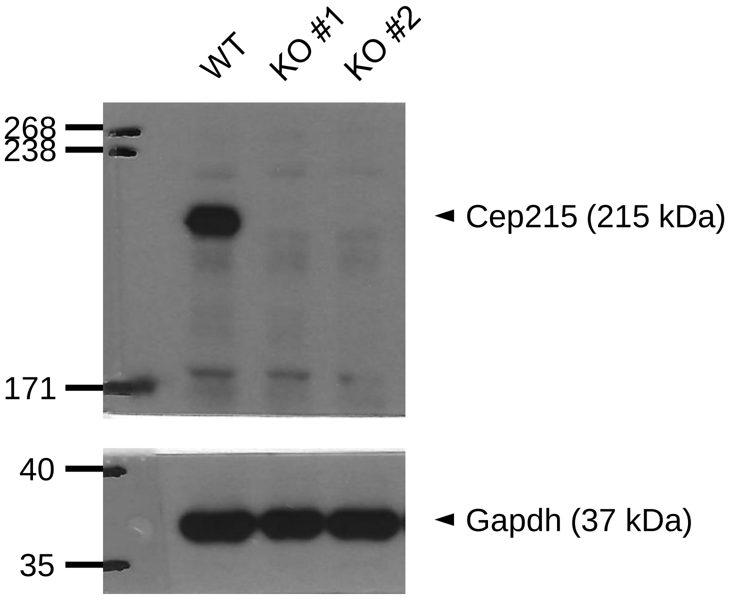

Supplementary Information 5. Full images of the immunoblots in Fig. 2f

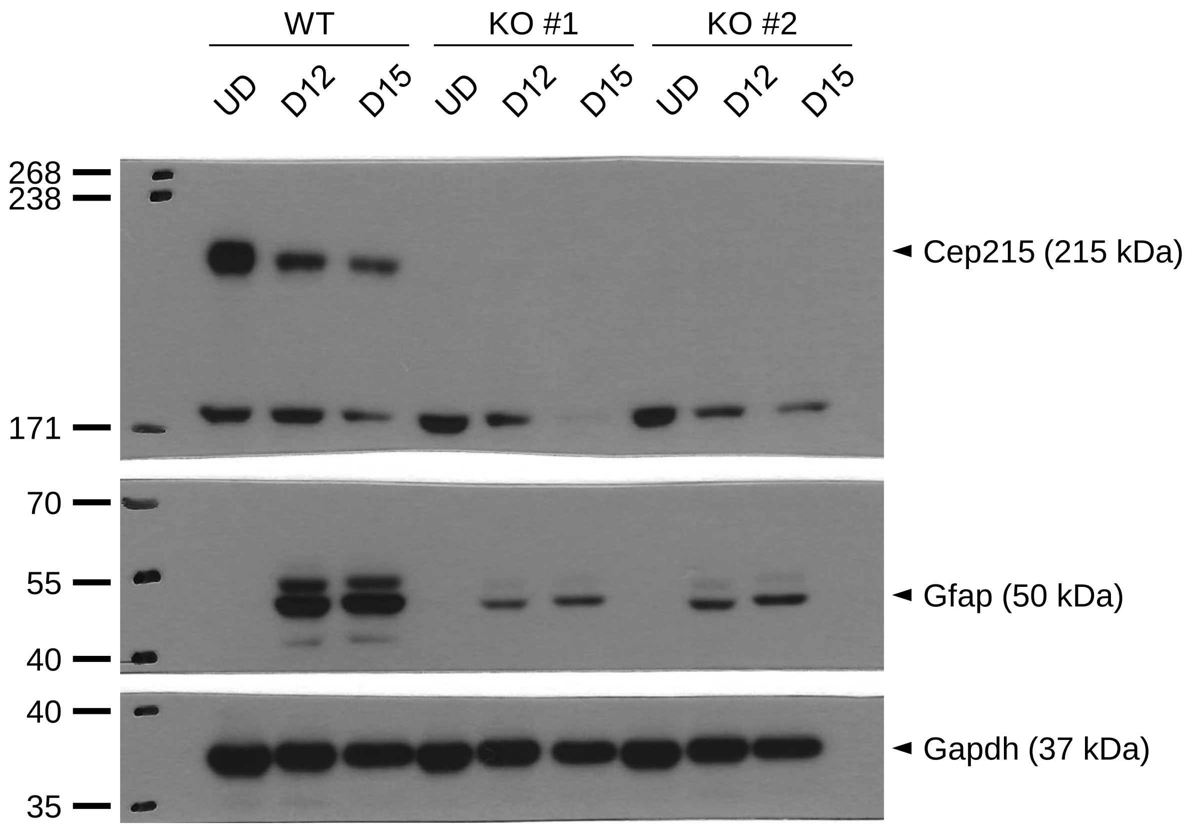

Supplementary Information 6. Full images of the immunoblots in Fig. 3c

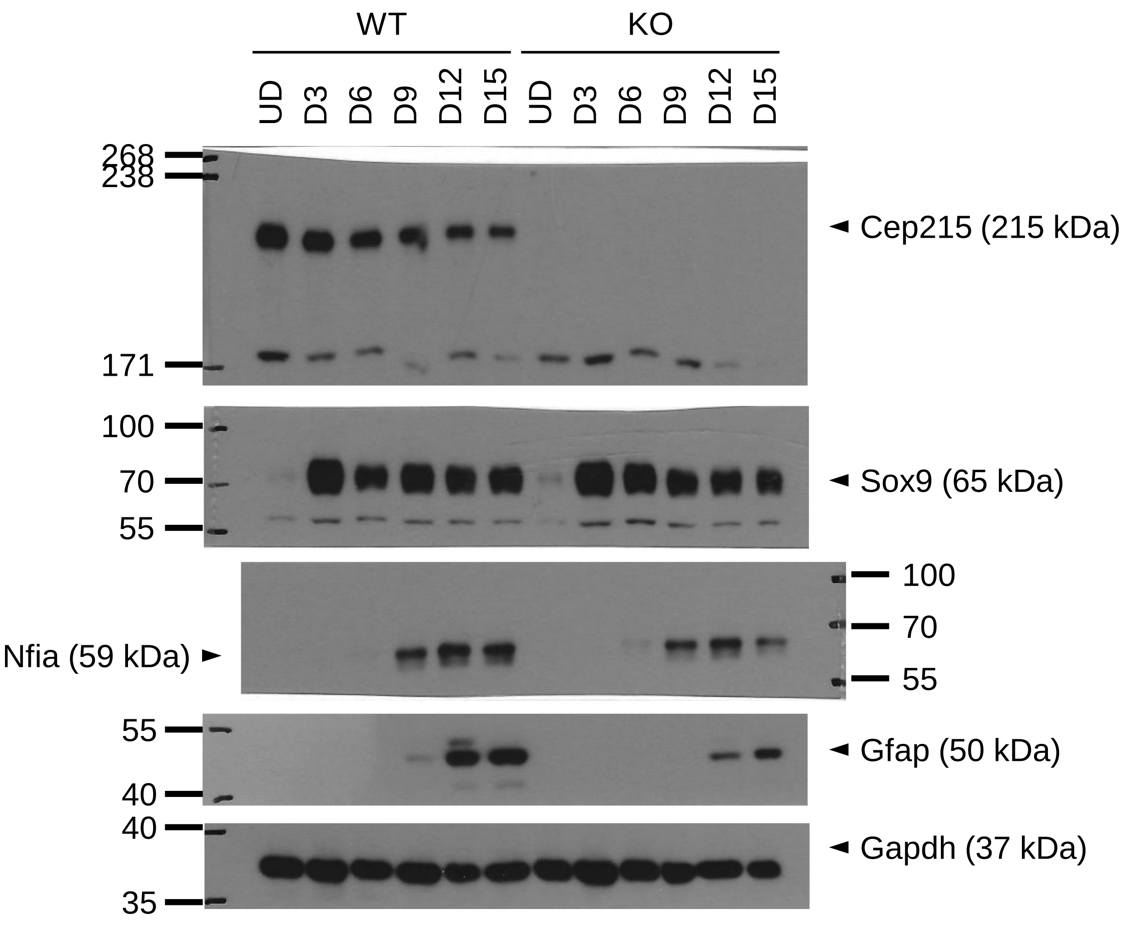

Supplementary Information 7. Full images of the immunoblots in Fig. 5a

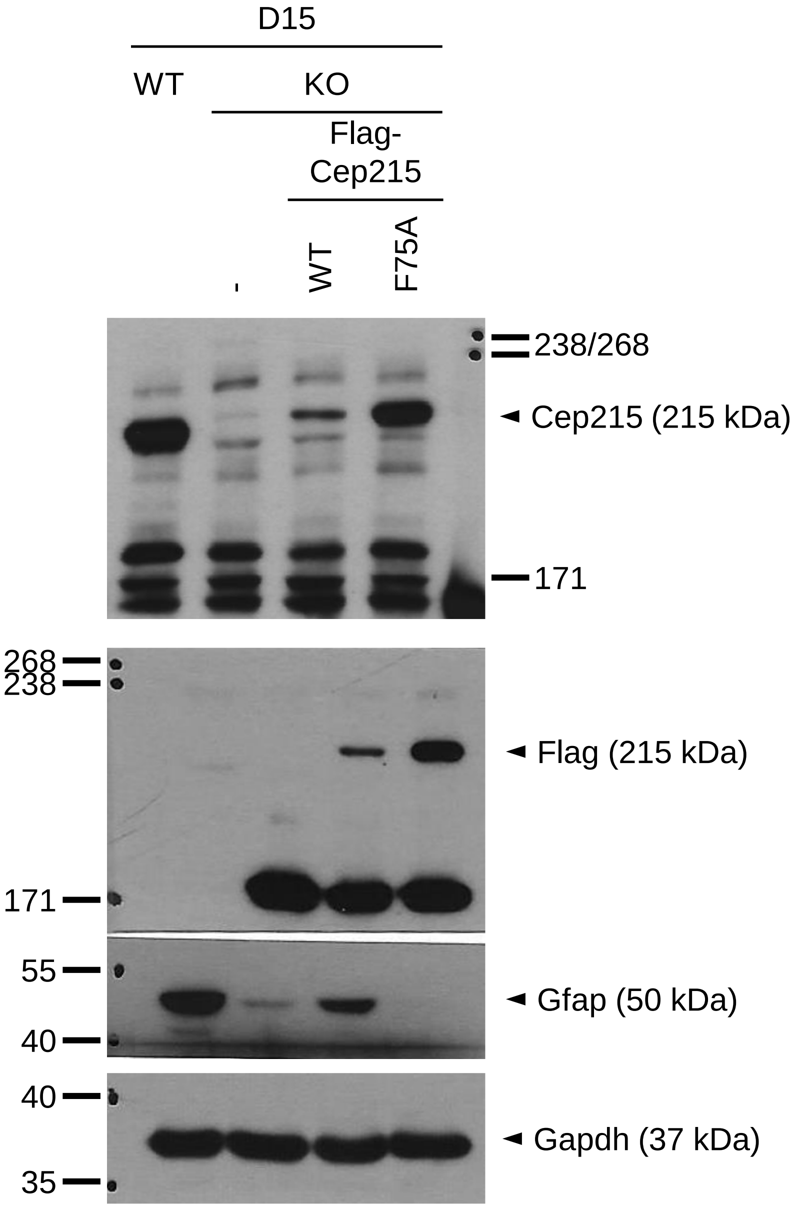

Supplement: Supplementary file 1 — Supplementary Information. [file 41598_2020_72728_MOESM1_ESM.pdf]
