# Supplementary figures and images for: Cep215 is essential for morphological differentiation of astrocytes
Source: Sci Rep. 2020 Oct 12;10:17000. doi: 10.1038/s41598-020-72728-7 (PMC7550586; doi:10.1038/s41598-020-72728-7)

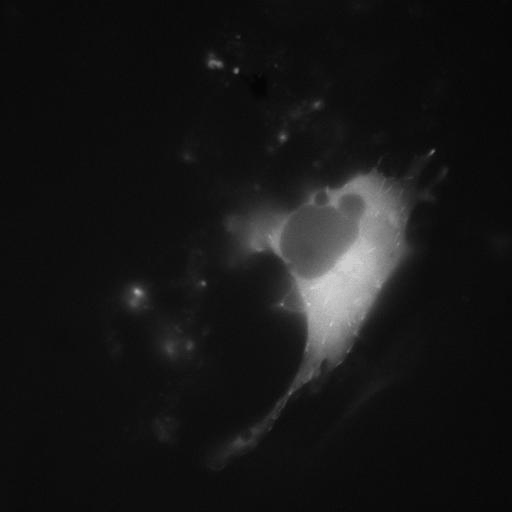

Supplement: Supplementary file 2 — Supplementary Information. [file 41598_2020_72728_MOESM2_ESM.gif]

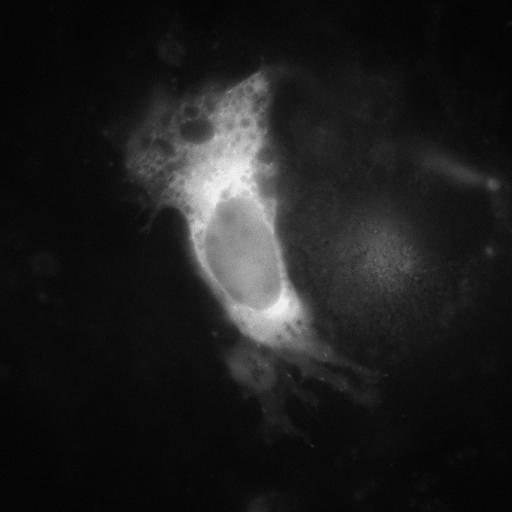

Supplement: Supplementary file 3 — Supplementary Information. [file 41598_2020_72728_MOESM3_ESM.gif]

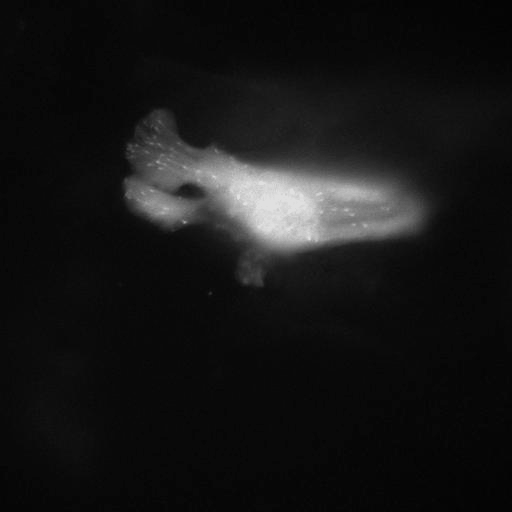

Supplement: Supplementary file 4 — Supplementary Information. [file 41598_2020_72728_MOESM4_ESM.gif]
